# Smartphone‐based scans of palate models of newborns with cleft lip and palate: Outlooks for three‐dimensional image capturing and machine learning plate tool

**DOI:** 10.1111/ocr.12859

**Published:** 2024-09-22

**Authors:** José Wittor de Macêdo Santos, Andreas Albert Mueller, Benito K. Benitez, Yoriko Lill, Prasad Nalabothu, Francisco Wilker Mustafa Gomes Muniz

**Affiliations:** ^1^ Department of Oral and Maxillofacial Surgery and Maxillofacial Prosthodontics School of Dentistry, Federal University of Pelotas Pelotas Rio Grande do Sul Brazil; ^2^ Department of Oral and Craniomaxillofacial Surgery University Hospital Basel and University Children's Hospital Basel Basel Switzerland; ^3^ Facial and Cranial Anomalies Research Group, Department of Biomedical Engineering and Department of Clinical Research University of Basel Basel Switzerland; ^4^ Department of Paediatric Oral Health and Orthodontics University Center for Dental Medicine UZB Basel Switzerland; ^5^ Department of Periodontology School of Dentistry, Federal University of Pelotas Pelotas Rio Grande do Sul Brazil

**Keywords:** cleft palate, computer simulations, dental technology, morphology, smartphone apps

## Abstract

**Objectives:**

To evaluate the performance of smartphone scanning applications (apps) in acquiring 3D meshes of cleft palate models. Secondarily, to validate a machine learning (ML) tool for computing automated presurgical plate (PSP).

**Materials and Methods:**

We conducted a comparative analysis of two apps on 15 cleft palate models: five unilateral cleft lip and palate (UCLP), five bilateral cleft lip and palate (BCLP) and five isolated cleft palate (ICP). The scans were performed with and without a mirror to simulate intraoral acquisition. The 3D reconstructions were compared to control reconstructions acquired using a professional intraoral scanner using open‐source software.

**Results:**

Thirty 3D scans were acquired by each app, totalling 60 scans. The main findings were in the UCLP sample, where the KIRI scans without a mirror (0.22 ± 0.03 mm) had a good performance with a deviation from the ground truth comparable to the control group (0.14 ± 0.13 mm) (*p* = .653). Scaniverse scans with a mirror showed the lowest accuracy of all the samples. The ML tool was able to predict the landmarks and automatically generate the plates, except in ICP models. KIRI scans' plates showed better performance with (0.22 ± 0.06 mm) and without mirror (0.18 ± 0.05 mm), being comparable with controls (0.16 ± 0.08 mm) (*p* = .954 and *p* = .439, respectively).

**Conclusions:**

KIRI Engine performed better in scanning UCLP models without a mirror. The ML tool showed a high capability for morphology recognition and automated PSP generation.

## 1 INTRODUCTION

The World Health Organization (WHO) has outlined recommendations for documentation and timing, advocating comprehensive registers.^1^ Among the primary methods of documenting cleft anatomy in three dimensions, models are predominantly obtained through impressions, which present the risk of material aspiration and airway obstruction.^2^ These issues add cumbersome logistics in treatment follow‐up and the use of pre‐surgical orthopaedic devices.^3^

In contrast, intraoral scanners offer a safe alternative, capable of producing high‐quality 3D models.^4^, ^5^ This digital approach facilitates the creation of PSP. Recently, intraoral scanning has become feasible in newborn cleft care, allowing a point‐of‐care digital workflow for 3D analysis and printing of presurgical appliances.^6^ Computer‐aided design and manufacturing (CAD‐CAM) of PSP have advanced significantly, with the use of an ML tool for automated landmarking prediction and PSP generation being the most innovative treatment workflow in cleft care.^7^

However, the main drawback of intraoral scanners lies in their cost, posing a barrier for implementation in many settings.^5^ Consequently, there is a need to explore mobile and cost‐effective tools including applications (app), and machine learning (ML) solutions for facial and intraoral capturing, particularly for applications in low‐ and middle‐income contexts.^8^ Recently, several studies have investigated smartphone scanning for medical applications in the face and head.^8^, ^9^, ^10^, ^11^, ^12^, ^13^ However, there are few studies investigating smartphone scan apps in individuals with cleft lip and palate, assessing ex vivo models and face scans.^8^, ^10^, ^14^ A single study in literature attempted palate 3D mesh creation from videos using complex computer engineering.^9^ Nevertheless, intraoral use was still impracticable due to conditions inside the mouth such as light, saliva, tissue texture and depth captured by smartphone cameras. Overall, especially with children, direct translation of smartphone‐based scanning for clinical applications might not be ethical, necessitating the use of mannequins,^11^ stone models^12^ or 3D‐printed models.^13^

This study aimed to evaluate the performance of two smartphone scanning apps compared to a control in acquiring 3D reconstructions of cleft palate models. Additionally, an ML tool for automated PSP generation on these meshes was validated. This is the first study to evaluate smartphone‐based scanning apps in palate models of newborns with cleft lip and palate, as well as the first to validate an ML for PSP generation using smartphone‐based scans. The hypothesis in this study was that smartphone apps have inferior performance compared to intraoral scanners but attain sufficient accuracy for recognition by an automated presurgical plate generator.

## 2 MATERIALS AND METHODS

### 2.1 Study design and ethical aspects

This was an in silico study wherein a sample of 3D‐printed models of individuals with cleft lip and palate underwent benchtop scanning by smartphone to allow mesh comparison and validation of an ML tool for automated PSP generation. The collection and further use of the clinical data received IRB approval by the Ethikkommission Nordwest‐ und Zentralschweiz (EKNZ) on 5 March 2020 (Protocol Number 2020‐00388).

### 2.2 Sample and power of the study

Fifteen patients with cleft lip and palate were selected by convenience from a pool of newborns whose intraoral scans (Medit i500, Medit Corp) of palate were available. Five patients for each type of cleft were included with the following criteria: complete unilateral or bilateral cleft lip and palate without Simonart's bands, and isolated cleft palate with no syndrome or other maxillofacial malformation. The clinical data of the patients were anonymized, and the scans were 3D printed for the study (Formlabs 3B, Formlabs Inc., Somerville, MA, USA). The goal was to consider the clinical applicability of smartphone‐based scanning specifically on newborn palates.

The power of the study was calculated a posteriori based on the comparisons for each app (with and without mirror) with the control group. Using a 95% confidence interval, mean and standard deviation for the main outcome a sample power of at least 95.58% was achieved (for the comparison between KIRI and the control group), which is considered appropriate.

### 2.3 Data collection strategy

#### 2.3.1 Smartphone device

Scans were performed using an iPhone 14 Pro Max, Apple Inc., equipped with a three‐camera system: a 48‐megapixel main camera with a 24 mm, ƒ/1.78 aperture, second‐generation sensor‐shift optical image stabilization, seven‐element lens; a 12‐megapixel ultra‐wide camera with a 13 mm, ƒ/2.2 aperture and 120° field of view; and a 12‐megapixel 2× telephoto camera enabled by a quad‐pixel sensor with a 48 mm, ƒ/1.78 aperture, second‐generation sensor‐shift optical image stabilization, and a seven‐element lens, featuring 100% Focus Pixels.

#### 2.3.2 Smartphone applications

Two scanning apps were assessed, namely Scaniverse (© 2024 Niantic, Inc.) and KIRI Engine pro version (© Kiri Innovations Science and Technology Inc.). Scaniverse, a free app, can perform scanning and generating 3D meshes by Light Detection and Ranging (LIDAR), photogrammetry and Gaussian splatting. In this study, the photogrammetry method for small objects was used, which captures images at 15 frames per second (fps) at the full resolution of the device. KIRI engine Pro version, a paid version, offers the same modes in addition to photogrammetry reconstruction by neural surface reconstruction (NSR) for featureless object scans, capturing images at 30 fps with 1080 pixels resolution. In this study, the latter was employed. Both apps' methods work in video‐capturing mode and are capable of scanning objects in a short time, simulating the principle of an intraoral scanner.

#### 2.3.3 Scanning protocol

For every 3D‐printed model, two scans were performed, one without and one with a mirror used for intraoral imaging to simulate the intraoral acquisition by a smartphone in a clinical setting. All scan acquisitions were systematically conducted by the same researcher (JWMS) under consistent light conditions in a controlled mini‐studio environment illuminated by five LED lamp strips (Foldio foldable studio) using the same mirror. The researcher was previously trained to perform the scanning process.

Each scan was conducted using the video‐capturing mode of the apps for 60 s. The models were positioned centrally on the plane surface of the mini‐studio on a white paper sheet against a black background to offset contrast. The scanning commenced from the superior (occlusal) view of the model using the smartphone. The device was then moved downward at an angle of 30–45° while capturing the maximum number of views around the model, maintaining a consistent distance of 20–30 cm. After this, scans were exported in stereolithography (stl) format for subsequent analysis and comparison with the control group.

#### 2.3.4 Control group

Fifteen additional scans of the same models were conducted using a professional intraoral scanner (Medit i500, Medit Corp), serving as the control group for comparison. These scans were overlapped with the original intraoral scans (ground truth‐GT) to validate their similarity and provide mean deviation (MD) values as references for comparison between groups.

### 2.4 In silico analysis (measurements)

#### 2.4.1 Mesh comparison

**FIGURE 1:**
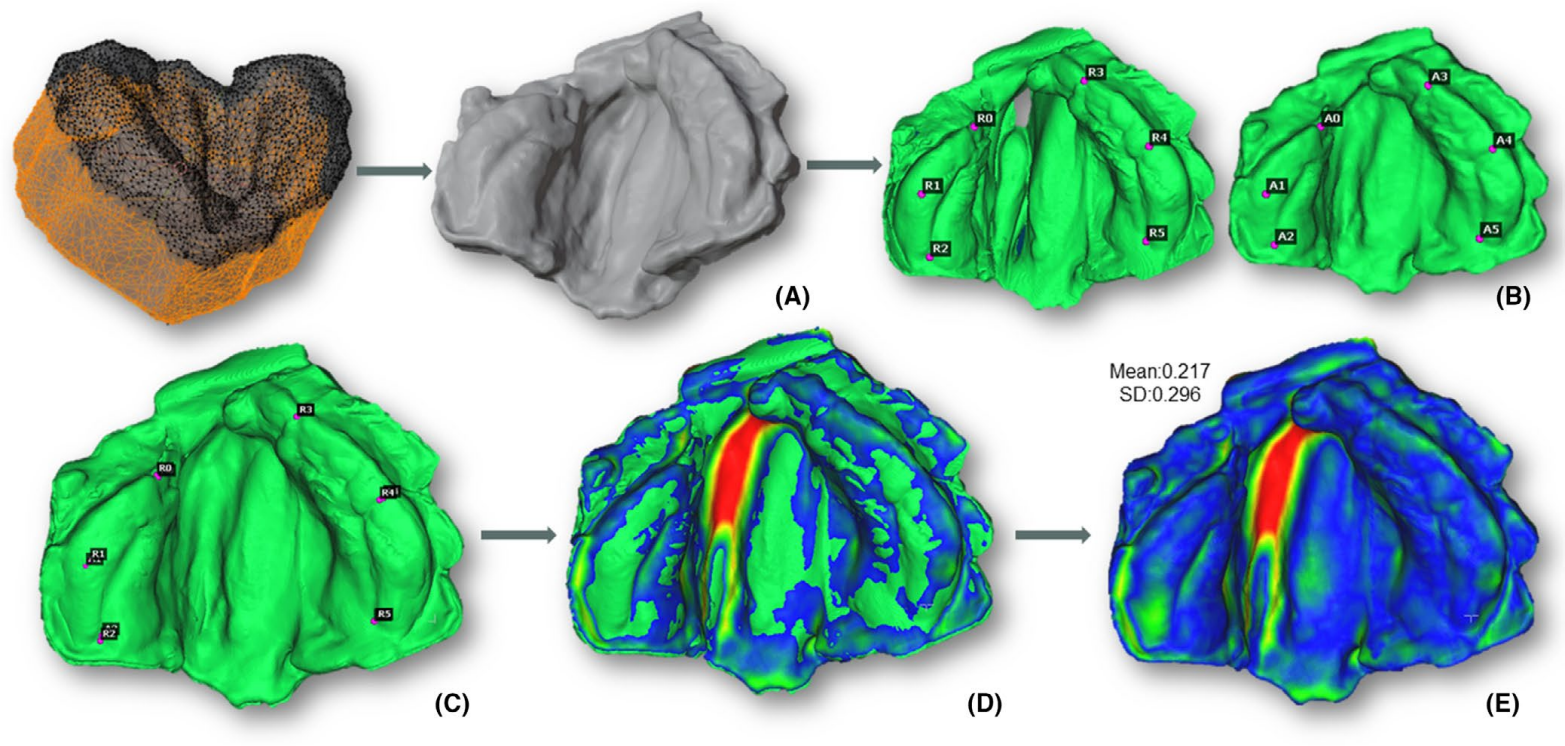
Procedure for mesh comparison. (A) Post‐processing and mesh cleaning in Blender. (B) Point matching alignment in CloudCompare. (C) Overlapping with rescaling of the mesh taking the reference scan. (D) Mesh distance computed. (E) Final distance‐to‐agreement histogram.

The post‐processing of the meshes in Blender (Blender, Blender Foundation)^15^ involved removing the unnecessary parts of the models while maintaining the palate and alveolar ridges. These models were then imported into the open‐source software for 3D point cloud processing, CloudCompare (version 2.12),^16^ where the qualitative and quantitative differences, including mean distance and standard deviation between the meshes, were calculated. Figure 1 depicts the post‐processing and mesh comparison.

In CloudCompare, all the smartphone meshes were aligned and rescaled to the reference mesh from the control group with simultaneous overlapping and comparison. For matching alignment, the same researcher with anatomy expertise (JWMS) manually selected six pairs of alveolar points on the two meshes, ensuring consistency by selecting these pairs as consistent anatomic points in all cases. The software then calculated a matrix minimizing the distance between the point pairs and aligned the meshes (Figure 1B). To improve the alignment accuracy, the iterative closest point (ICP) algorithm function of the software was applied.

**FIGURE 2:**
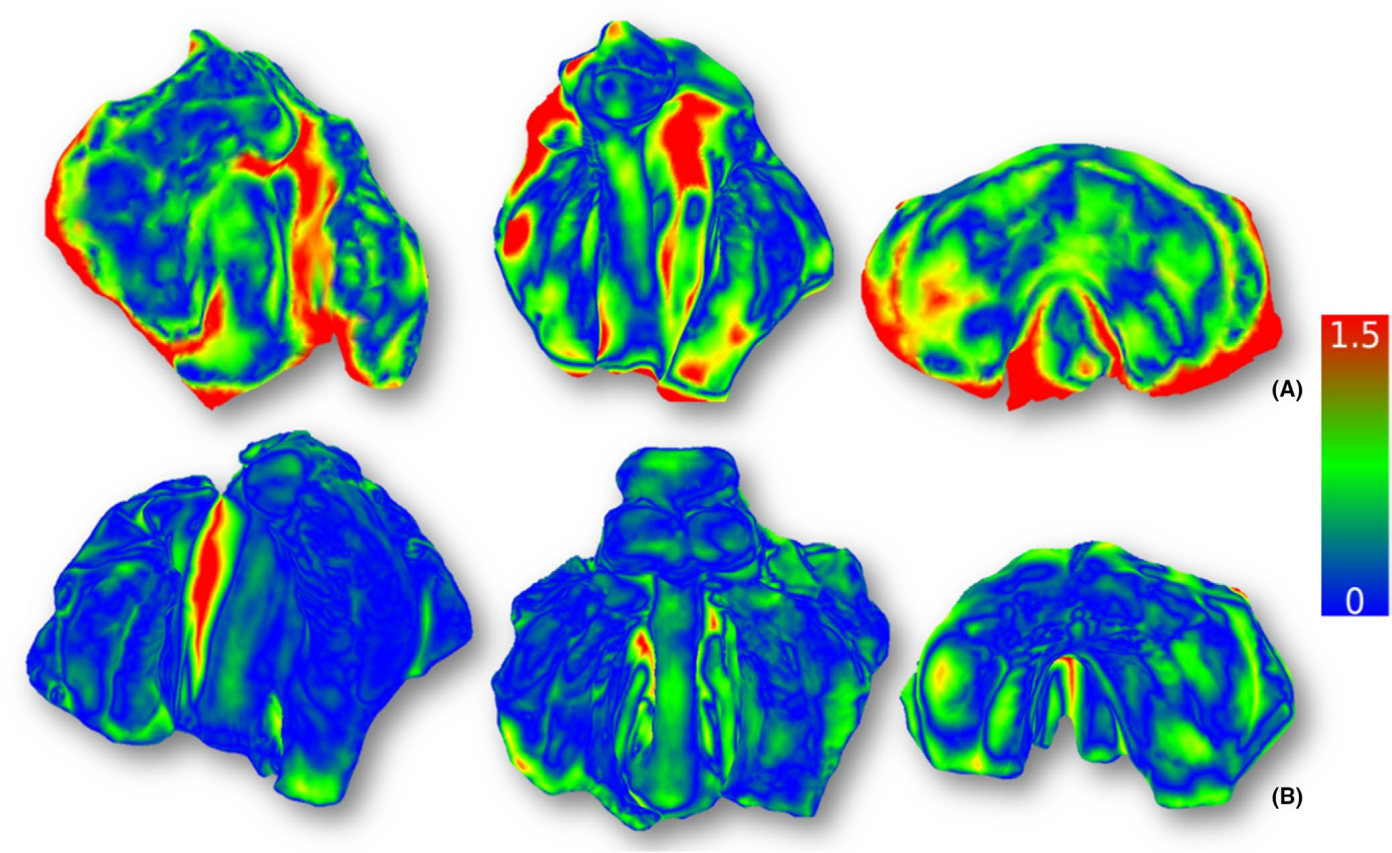
Distance‐to‐agreement histogram (0–1.5 mm) showing (A) lower‐quality scans and (B) optimal‐quality scans after mesh comparison in CloudCompare.

As a final step, the function ‘Compute cloud/mesh distance’ was applied, and the mean deviation (MD) and standard deviation (SD) between the meshes were recorded in millimetres (mm) for each model, serving as the primary outcome (Figure 1E). Qualitative results of the scan comparison were created using distance‐to‐agreement histograms (heat map‐like) with shades ranging from red to blue, indicating the extremes of the deviation values from the least (1.5 mm) to the best (0 mm) matching, respectively (Figure 2). This methodology follows previous studies with similar purposes and use of the same software.^11^, ^12^, ^13^, ^17^

#### 2.4.2 Machine learning tool for automated PSP generation

**FIGURE 3:**
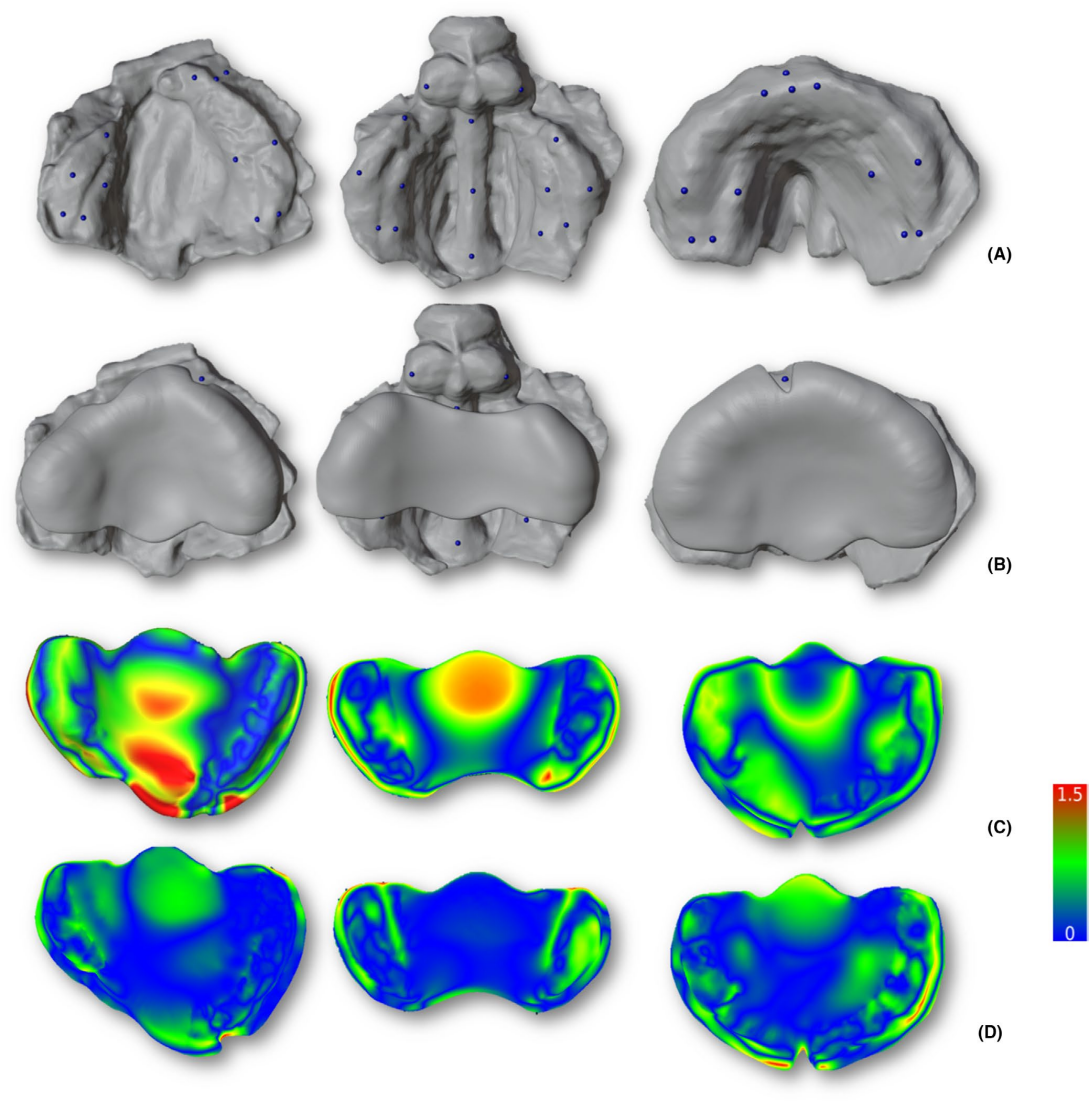
Automated presurgical plate generation. (A) Automated anatomically‐based landmarks on unilateral and bilateral complete cleft lip and palate, with the isolated cleft palate cases needing manual landmarking. (B) Different automatically generated presurgical plate meshes for each cleft type, allowing manufacturing by 3D printing. (C) Distance‐to‐agreement histogram (0–1.5 mm) showing lower‐quality plates and (D) optimal‐quality plates after mesh comparison in CloudCompare.

**FIGURE 4:**
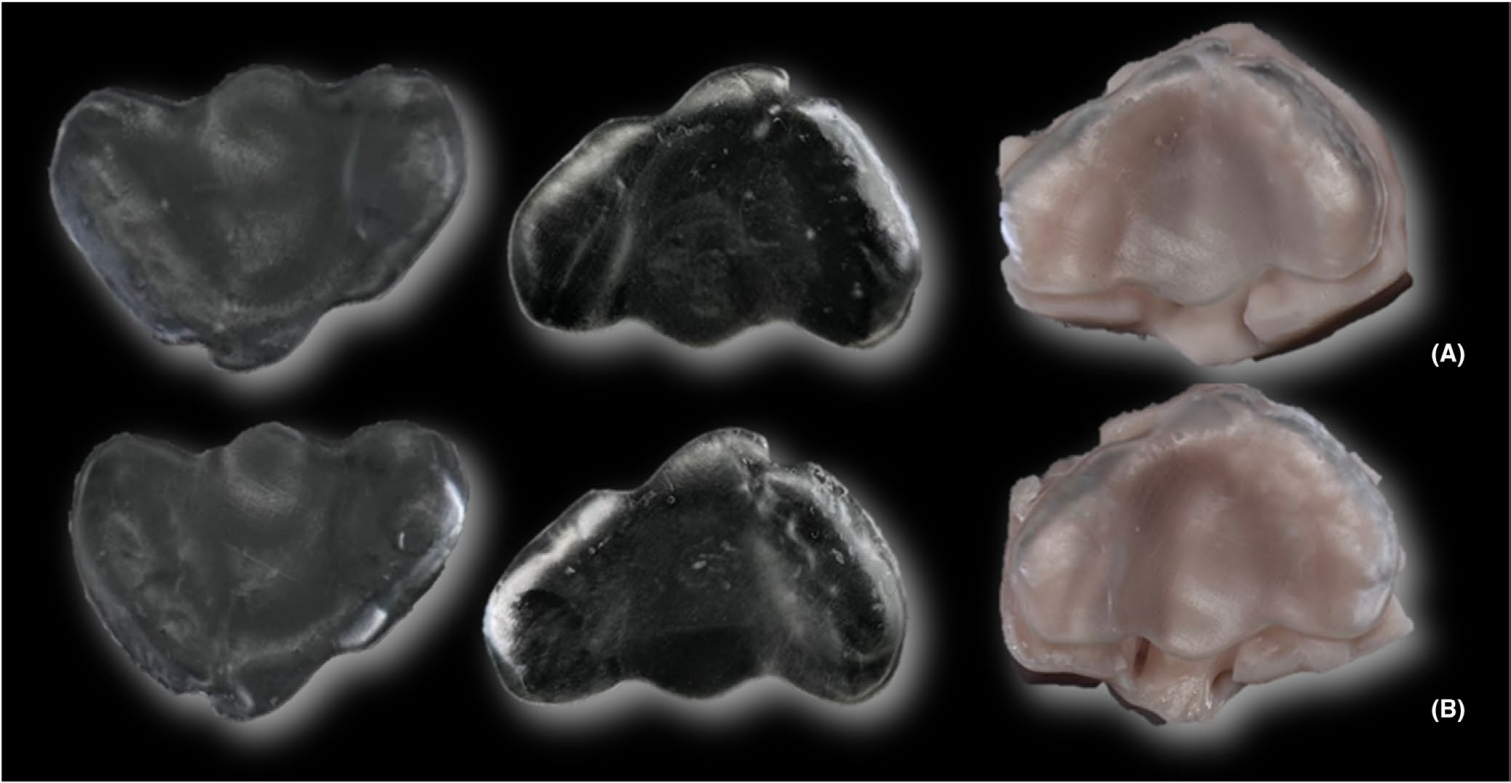
Models and presurgical plate physical comparison. (A) One optimal model acquired after smartphone scanning and its presurgical plate at the top row. (B) Corresponding ground truth model and presurgical plate at the bottom.

An ML tool was assessed for its capability for morphology recognition and automated PSP generation using smartphone scans as inputs. The tool, hosted as a plugin to open software for 3D designing (Blender), was developed using a trained DiffusionNet model that predicts anatomically‐based landmarks and creates an automatically generated PSP (Figure 3A,B). The complete method may be found elsewhere.^7^ Subsequently, all plate meshes were analysed using the same methodology as the models. The comparison was made between each mesh and the corresponding control plate (Figure 3C,D). One of the best‐acquired models and its generated PSP among the sample were 3D‐printed for physical evaluation and comparison with the correspondent GT by two senior craniomaxillofacial surgeons with clinical experience in the use of PSP devices (BioMed Clear, Formlabs Inc., Somerville, MA, USA) (Figure 4).

### 2.5 Statistical analysis

Statistical analysis involved comparative assessment between the groups. Descriptive statistics were used to express quantitative variables as mean ± SD, and mean differences with their distributions between the groups were assessed. The distribution of data symmetry was tested using a Kolmogorov–Smirnov test, followed by one‐way anova. To identify significant differences between groups, the post‐hoc Tukey test was used. The *p* value was calculated to find statistically significant differences. The analysis was performed using the Statistical Package for Social Sciences Software version 29 (SPSS Inc., Chicago, IL). Statistical significance was established as *p* < .05. Independent analyses were conducted for the whole sample and for each group of cleft (UCLP, BLCP, and ICP). Analyses were also conducted for the models and their corresponding plates.

## 3 RESULTS

All 15 models were successfully scanned without and with a mirror using both apps, resulting in a total of 30 3D scans acquired by each app, totalling 60 scans. For the meshes' distance outcome, data were considered skewed (*p* = .084). Moreover, all the scans of the control group showed fidelity to the original intraoral scans (0.09 ± 0.10 mm), validating their use as controls.

**FIGURE 5:**
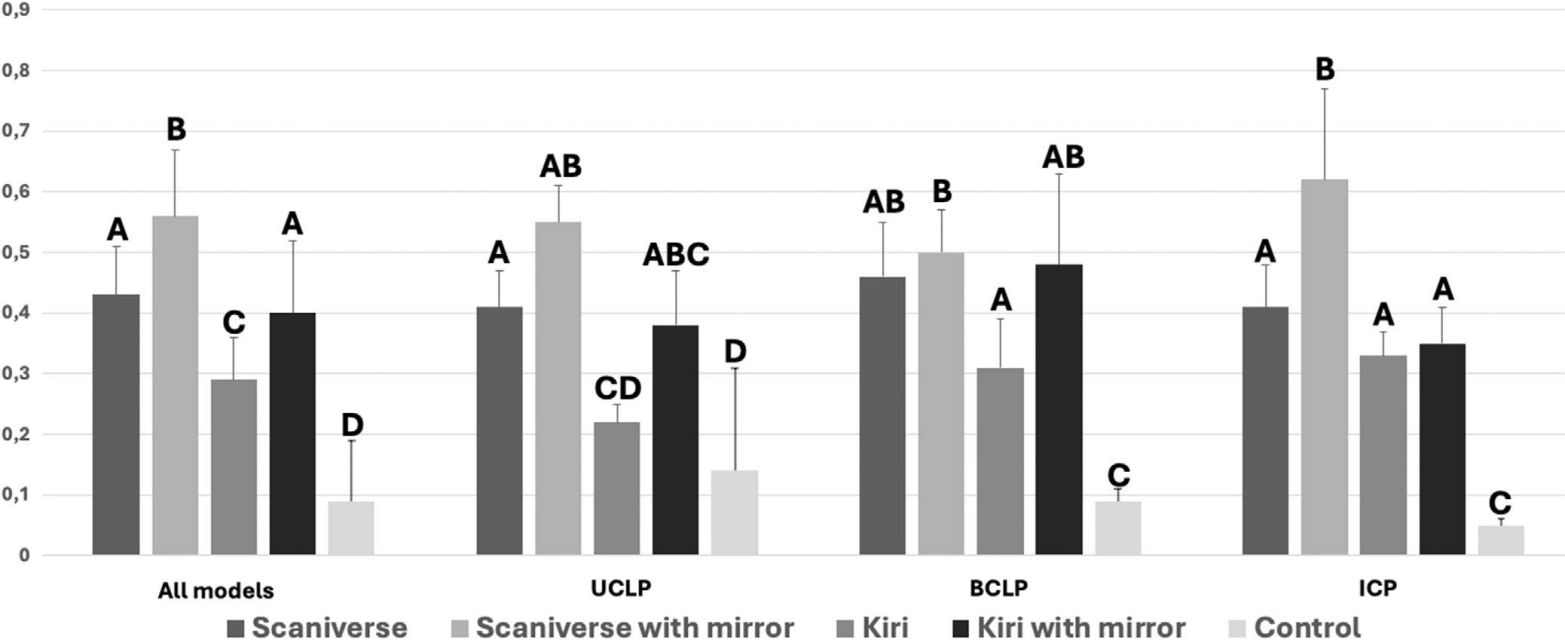
Graphic highlighting the group comparison of all models and of each cleft group (unilateral cleft lip and palate; complete bilateral cleft lip and palate; and isolated cleft palate). Different letters mean statistically significant differences between groups in each analysis (*p* < .05).

Both apps demonstrated inferior performance compared to controls, except in the UCLP sample where the KIRI scans without a mirror (0.22 ± 0.03) demonstrated performance statistically similar to the control group (0.14 ± 0.17) (*p* = .653). In contrast, Scaniverse scans with a mirror exhibited the lowest accuracy across all comparisons. In all the samples (UCLP, BCLP and ICP), KIRI scans with a mirror yielded similar results to Scaniverse without a mirror. The results and statistically significant differences are depicted in Figure 5, where group bars with different letters indicate statistically significant differences between groups in the pairs analysis, and intersections or no differences between groups are shown with double or triple letters.

The ML tool successfully predicted landmarks and automatically generated plates in all the models and types of clefts, except ICP. The inability to generate plates in the ICP group stemmed from a lack of training of the ML tool on data specific to this type of cleft. Therefore, data for this cleft group was not analysed for the plate outcome.

**FIGURE 6:**
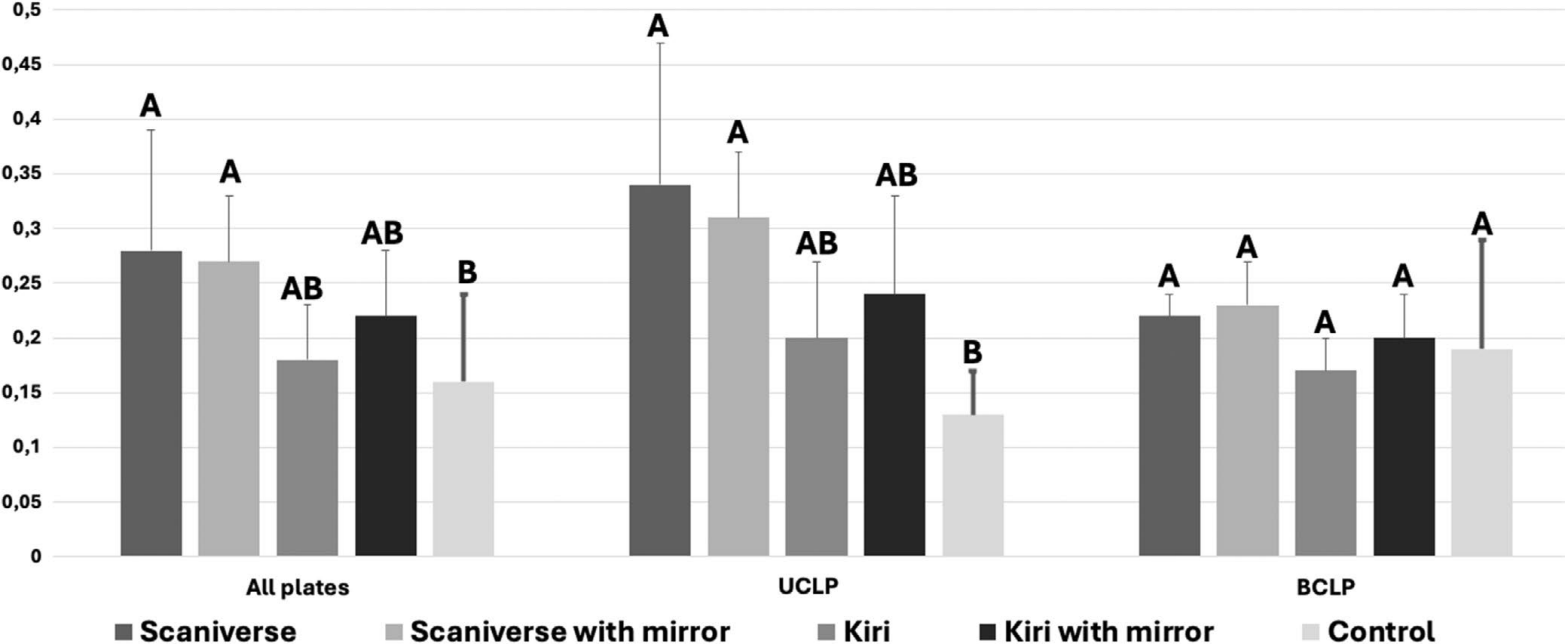
Graphic highlighting the group comparison of all plates (except for isolated cleft palate) and by each cleft group (unilateral and bilateral cleft lip and palate). Different letters mean statistically significant differences between groups in each analysis (*p* < .05).

In the comparison of all plates, a skewed data distribution was also detected (*p* = .097). KIRI exhibited statistically significant superiority in performance with (0.22 ± 0.06 mm) or without a mirror (0.18 ± 0.05 mm) compared to Scaniverse, being comparable with controls (0.16 ± 0.08). In subgroup analyses, KIRI maintained significantly better performance than Scaniverse in UCLP, presenting no statistically significant differences with the control group. Conversely, in the BCLP group, all plates exhibited no statistically significant differences (Figure 6).

## 4 DISCUSSION

While the process of scanning, exporting and post‐processing to produce a 3D model involves multiple steps when using smartphones, the use of a professional scanner yields high‐quality models rapidly, suitable for various applications.^12^ However, the portability, low cost and availability of smartphones make them a viable option for 3D capturing, particularly in low‐ and middle‐income settings.^9^, ^17^ Moreover, smartphone scanning is a rapid tool, with our study demonstrating that a standard scan time of 60 s per scan might be sufficient, since there was no significant difference in the quality of scans acquired by short videos (45 s) or long videos (120 s).^11^ This facilitates quick and easy scanning, especially important for application in children.

The selection of the specific scanning modes and apps of this study (KIRI and Scaniverse) was based on their true scanning/video capturing mode, which allows for automated photogrammetry reconstruction. LIDAR scanning is known to be unsuitable for small objects. The professional intraoral scanner was chosen for the control group due to its established depth of field and being the current clinical standard in cleft care as documented in specialized literature.^5^

Several studies have evaluated smartphone scan apps for various purposes in dentistry and medicine.^10^, ^12^, ^17^, ^18^, ^19^, ^20^ The direct clinical application can be impracticable, necessitating ex vivo studies for gaining understanding of the tool and application. Moreover, ethical considerations mandate the use of models or mannequins for the simulation and assessment of smartphone scans, particularly in children.^13^, ^14^ Despite these limitations, real‐life applications in clinical practice have shown promise for ear reconstruction,^21^ dentistry planning^22^ and cleft lip measurements.^8^

Analysis of our heat maps revealed that smartphone scanning is most accurate on the prominent ridge segments, while less accurate on small surface details and deeper curved areas (cleft, palatine rugae and grooves), reflecting the known limitations of smartphone scanning in capturing complex anatomy.^9^, ^18^, ^20^ Notwithstanding, the KIRI Engine pro version is advertised as having an improved photogrammetry reconstruction algorithm, its performance was statistically significant superior only in the UCLP group without a mirror. The use of a mirror reduced the performance, with Scaniverse having the lowest‐quality scans. This limitation has been previously described.^9^

It is believed that the studio was used to simulate the light conditions in clinical settings appropriately, as LED lights are commonly used in dental chairs and surgery theatres. KIRI Engine allows uploading videos recorded out of the app, directly by the smartphone camera at 4 K resolution, which theoretically can improve 3D reconstructions quality with more points and vertices.^11^ This function was not explored to avoid bias in comparison with Scaniverse. Other apps are limited to photo‐by‐photo acquisition which considerably increases the time of acquisition since some apps indicate the need for >70 pictures for the creation of a mesh with good quality.

Smartphone's popularity and availability make it an attractive device for scanning.^8^ However, the assessment of more smartphone apps and different mobile technologies across a large infant sample is necessary to evaluate the feasibility of the adapted methodology proposed in this study. This study sets an initial milestone for evolving smartphone‐based technology for the cleft field. Future studies may concentrate on non‐commercial tools for acquisition and 3D reconstruction with broad compatibility to assess its validity for clinically transferable results and allow widespread clinical use.

The ML tool for PSP effectively identifies UCLP and BCLP model meshes from smartphone scan apps. It accurately predicted the landmarks and generated printable plate meshes automatically. This is an important validation of the tool previously applied only on palatal meshes obtained by professional intraoral scanner.^7^ While manual landmarking was required for ICP, the tool was still capable of automated PSP generation. The adaptability of the tool to diverse inputs underscores its robustness and promising applicability. A plate generated from an optimal scan even achieved comparable fitting to the GT correspondent model on physical evaluation, highlighting that the method comes within reach for clinical translation.

### 4.1 Limitations

Despite a systematic acquisition protocol, operator and phone movement during image capture could introduce inaccuracies and artefacts. The distance between the camera and the target also affects accuracy and image depth capturing, necessitating skilled operator attention.^12^, ^17^ The findings remain therefore restricted to the in silico environment and cannot be generalized for clinical settings.

### 4.2 Strengths

This study is the first to evaluate cleft palate 3D capturing by smartphone apps and validate a machine learning PSP generator using smartphone‐based scans. While advancements in mobile technologies are evolving rapidly, our study demonstrates their realistic potential for future intraoral 3D acquisition for cleft care.

## 5 CONCLUSIONS

- KIRI Engine showed superior performance compared to Scaniverse, with statistically significant similarity to the control group scanning UCLP models without a mirror.
- The ML tool demonstrated high morphology recognition capability, generating automated PSP in all UCLP and BCLP cases.
- Although still challenging in the clinical applications, the in silico results of smartphone‐based scans show potential for clinical transfer.

## AUTHOR CONTRIBUTIONS

Conceptualization: José Wittor de Macêdo Santos, Andreas Albert Mueller, Prasad Nalabothu and Francisco Wilker Mustafa Gomes Muniz; Methodology: José Wittor de Macêdo Santos, Andreas Albert Mueller, Prasad Nalabothu and Francisco Wilker Mustafa Gomes Muniz; Investigation: José Wittor de Macêdo Santos, Yoriko Lill and Prasad Nalabothu; Validation: Benito K. Benitez, José Wittor de Macêdo Santos, Yoriko Lill and P.N; Software: Benito K. Benitez, José Wittor de Macêdo Santos, Yoriko Lill and Prasad Nalabothu; Formal Analysis: José Wittor de Macêdo Santos, Andreas Albert Mueller and Benito K. Benitez; Data Curation: José Wittor de Macêdo Santos and Francisco Wilker Mustafa Gomes Muniz; Writing – Original Draft Preparation: José Wittor de Macêdo Santos, Prasad Nalabothu and Francisco Wilker Mustafa Gomes Muniz; Writing – Review And Editing: José Wittor de Macêdo Santos, Benito K. Benitez, Yoriko Lill, Prasad Nalabothu and Francisco Wilker Mustafa Gomes Muniz; Supervision: Andreas Albert Mueller, Benito K. Benitez, Yoriko Lill, Prasad Nalabothu and Francisco Wilker Mustafa Gomes Muniz All authors have read and agreed to the published version of the manuscript.

## FUNDING INFORMATION

This study was supported by the Basel Research Centre for Child Health (BRCCH), supporting the Research Consortium for Pediatric Digital Health with a Multi‐Investigator Project ‘Burden‐Reduced Cleft Lip and Palate Care and Healing’, led by Principal Investigator A.A.M. The work was further supported by the Swiss National Science Foundation under grant no. 205321_205008. This study was also supported by the Coordenação de Aperfeiçoamento de Pessoal de Nível Superior – Brasil (CAPES) – Finance Code 001.

## CONFLICT OF INTEREST STATEMENT

The authors have no conflict of interest to declare neither any link with companies. All authors have viewed and agreed to the submission.

## ETHICS STATEMENT

The collection and further use of the clinical data received IRB approval by the Ethikkommission Nordwest‐ und Zentralschweiz (EKNZ) on 5 March 2020 (protocol number 2020‐00388).

## Data Availability

The data underlying this article will be shared on reasonable request to the corresponding author.
